# A preliminary clinical study related to vestibular migraine and cognitive dysfunction

**DOI:** 10.3389/fnhum.2024.1512291

**Published:** 2024-12-23

**Authors:** Tingting Sun, Yake Lin, Yanan Huang, Yonghui Pan

**Affiliations:** The First Affiliated Hospital of Harbin Medical University, Harbin, Heilongjiang, China

**Keywords:** vestibular migraine, cognitive dysfunction, vestibular function, canal paresis (CP) values, anxiety and depression

## Abstract

**Background and purpose:**

Vestibular migraine (VM) is a common clinical disorder with a genetic predisposition characterized by recurrent episodes of dizziness/vertigo. Patients often complain of the presence of cognitive dysfunction manifestations such as memory loss, which causes great distress in daily life. In this study, we will explore the characteristics and possible risk factors of VM-related cognitive dysfunction by observing the cognitive function and vestibular function status of VM patients, laying the foundation for further exploration of the mechanisms of VM-related cognitive dysfunction.

**Methods:**

This study included 61 patients with VM and 30 healthy individuals matched for age, gender, and education level. All subjects underwent the Addenbrooke’s Cognitive Examination-Revised (ACE-R), Dizziness Handicap Inventory (DHI), Hospital Anxiety and Depression Scale (HADS), Patient Health Questionnaire-9 (PHQ-9), and Generalized Anxiety Disorder-7 (GAD-7) at the first time of enrollment. Based on the ACE-R scores, the VM group was divided into the VM with cognitive dysfunction (VM-CogD) group (ACE-R < 86) and the VM without cognitive dysfunction (VM-NoCogD) group (ACE-R ≥ 86). The VM-CogD group was further categorized based on DHI scores into mild, moderate, and severe dizziness/vertigo subgroups (DHI ≤ 30 for mild, 30 < DHI ≤ 60 as moderate, and DHI > 60 as severe). All subjects underwent the head-shaking test, head-impulse test, test of skew, Romberg test, Unterberger test, videonystagmography, and caloric test to evaluate their vestibular function including the semicircular canals, vestibulo-ocular reflex pathway, and vestibulo-spinal reflex pathway. Differential analysis, correlation analysis, and ROC curve analysis were used to analyze the characteristics and influencing factors of the above clinical indicators in VM patients. It was considered that *p*-value < 0.05 was statistically significant, and | r| > 0.3 indicated a good correlation.

**Results:**

There were no significant differences between the VM group and healthy control (HC) group in sex, age and education level. The total ACE-R score of the VM group was [82 (68.5, 87)], and the total ACE-R score, memory, verbal fluency, language, and visuospatial function scores were significantly lower than those of the HC group (*p*-value < 0.05) The percentage of horizontal semicircular canal dysfunction in the VM group (82.0%), the percentage the ocular motor dysfunction (49.2%), the positive percentage of the head-shaking test (27.9%), head-impulse test (37.7%), Romberg’s sign (60.7%), and Unterberger’s sign (60.7%) were significantly higher than those in the HC group (*p*-value < 0.05). Comparing the VM-CogD group (ACE-R < 86) with the VM-NoCogD group (ACE-R ≥ 86), the differences in Canal Paresis (CP) value, age, years of education, and duration of the disease were statistically significant (*p*-value < 0.05). In the VM-CogD group, CP value was negatively correlated with the ACE-R total score (*r* = 0.571, *p*-value = 0.000), memory (*r* = 0.526, *p*-value = 0.000), verbal fluency (*r* = 0.345, *p*-value = 0.024), language (*r* = 0.524, *p*-value = 0.000), and visuospatial function (*r* = 0.340, *p*-value = 0.026) scores. Age was negatively correlated with language functioning scores (*r* = 0.384, *p*-value = 0.011), and years of education was positively correlated with ACE-R total score (*r* = 0.504, *p*-value = 0.001) and language functioning (*r* = 0.455, *p*-value = 0.002) scores. When the cutoff values for the CP value, age, years of education, and duration of disease were 25.5, 33, 15.5, and 6.5, the accuracy of predicting VM-related cognitive dysfunction was the highest. The differences in DHI, DHI-P, DHI-E, DHI-F, HADS, PHQ-9, and GAD-7 scores were statistically significant between the VM group and the HC group (*p*-value < 0.05). In VM-CogD patients presenting with moderate dizziness/vertigo, the DHI total score was negatively correlated with the ACE-R total score (*r* = 0.539, *p*-value = 0.008), the DHI-F score was negatively correlated with the language (*r* = 0.450, *p*-value = 0.031) and visuospatial functioning part (*r* = 0.415, *p*-value = 0.049) scores, and the HADS-D scores were negatively correlated with the ACE-R total score and the part of memory functioning score (*r* = 0.539, *p*-value = 0.008).

**Conclusion:**

(1) VM Patients exhibit multifaceted vestibular dysfunction and varying degrees of cognitive dysfunction, and cognitive function is affected by age, duration of illness, years of education, and vestibular function; (2) VM is a functional disorder, and the function disturbance, in conjunction with anxiety and depression, may participate in the occurrence of development of cognitive dysfunction in VM.

## 1 Introduction

VM is one of the most common disorders with episodic vestibular syndrome in clinical practice, predominantly affecting young to middle-aged women. It is primarily characterized by moderate to severe dizziness or vertigo, with or without unilateral pulsating headache, photophobia, phonophobia, and visual aura. Headache is not specific to VM, as patients may never experience it. However, when it does occur, it can be before, after, or even during the vestibular symptoms (Lampl et al., 2019). In addition to the typical dizziness and headache symptoms, there are some accompanying symptoms such as brain fog and difficulty finding words (Beh et al., 2019). In recent years, numerous studies have found that VM patients experience cognitive dysfunction, involving various aspects like attention, memory, visuospatial function, language function, etc (Balci et al., 2018; Preysner et al., 2022; Wang et al., 2016). It was also found that the cognitive dysfunction in VM patients are related to the duration of the disease and the frequency and the severity of symptom (Lu et al., 2024). But it was neglected because of the youthful onset and mild dysfunction of cognitive (Çelebisoy et al., 2022). The recurrent symptoms of dizziness and headache have already influenced the efficiency of work and daily life for the patients (Molnár et al., 2022). The appearance of cognitive dysfunction symptoms further exacerbates this suffering. Therefore we should pay attention to this issue, which is to identify VM-related cognitive dysfunction early and take appropriate measures.

There are many risk factors for the occurrence and progression of cognitive dysfunction, including age, level of education, psychological and emotional factors, traumatic brain injury, white matter lesions, and underlying conditions such as diabetes and coronary artery disease (Ding et al., 2019; Hugo and Ganguli, 2014). Olfactory dysfunction is also closely associated with cognitive dysfunction (Molnár et al., 2023b). Current studies have found that vestibular function is closely related to cognitive function, while it is particularly evident in vestibular diseases (Bigelow and Agrawal, 2015; Rizk et al., 2020). Patients with unilateral or bilateral vestibular dysfunction exhibit impairments in visuospatial function, memory, attention and executive function. The more severe the vestibular function damage, the more pronounced the cognitive impairment (Popp et al., 2017); Patients with vestibular neuritis also show varying degrees of impairment in executive function (Moser et al., 2017), visuospatial perception, and visuospatial memory function (Oh et al., 2023). Eraslan Boz et al. (2023) found that patients with Ménière’s disease experience widespread cognitive dysfunction, including in memory, attention, executive functioning, and visuospatial functioning. Many studies have identified varying degrees of vestibular dysfunction in VM patients (Yujie et al., 2018). However, whether vestibular dysfunction in VM patients is the cause of cognitive dysfunction and the specific interaction mechanism between the two is unclear. This study aims to investigate and analyze the vestibular and cognitive functions in VM patients to elucidate the correlation and the underlying mechanisms, providing new insights for the early identification and intervention of VM-related cognitive dysfunction.

## 2 Materials and methods

### 2.1 Participants

This study was conducted from February 2023 to December 2023. The study was approved by the Ethics Committee of the First Affiliated Hospital of Harbin Medical University, and all participants voluntarily participated with signed informed consent forms. All 61 VM patients were recruited from the Dizziness and Vertigo Clinic of the Neurology Department at the First Affiliated Hospital of Harbin Medical University. Meanwhile the control group which include 30 participants, matched for age, gender and education years, required no history of headaches, dizziness/vertigo, or other serious illnesses and they mostly consisted of family members or friends accompanying the outpatients. The inclusion and exclusion criteria for VM patients are as follows. Upon enrollment, VM patients were assessed using the ACE-R, DHI, HADS, PHQ-9, and GAD-7 scales. Based on ACE-R scores, VM patients were divided into two groups: the VM-CogD group (ACE-R < 86) and the VM-NoCogD group (ACE-R ≥ 86). The VM-CogD group was further subdivided into 3 groups based on their total DHI score: mild, moderate, and severe dizziness/vertigo with cognitive dysfunction (DHI ≤ 30 for mild, 30 < DHI ≤ 60 for moderate, and DHI > 60 for severe). Bedside and laboratory vestibular function examinations, including the head-shaking test, head-impulse test, test of skew, Romberg test, Unterberger test, videonystagmography, and caloric test were performed on VM patients during the remission phase of episodes. All examinations were conducted by the same neurophysiologist.

#### 2.1.1 Inclusion criteria

(1) Meet the diagnostic criteria for VM according to the Bárány Society (Table 1) (Lempert et al., 2012); (2) Be able to read and complete questionnaires; (3) Be able to communicate properly; (4) Sign the informed consent form.

**TABLE 1:** Diagnostic criteria of VM.

VM	Possible VM
A. At least 5 episodes with vestibular symptoms of moderate or severe intensity, lasting 5 min to 72 h	A. At least 5 episodes with vestibular symptoms of moderate or severe intensity, lasting 5 min to 72 h
B. Current or previous history of migraine with or without aura according to the International Classification of Headache Disorders (ICHD)	B. Only one of the criteria B and C for vestibular migraine is fulfilled (migraine history or migraine features during the episode)
C. One or more migraine features with at least 50% of the vestibular episodes: a. Headache with at least two of the following characteristics: one-sided location, pulsating quality, moderate or severe pain intensity. b. Aggravation by routine physical activity c. Photophobha and phonophobia	C. Not better accounted for by another vestibular or ICHD diagnosis
D. Not better accounted for by another vestibular or ICHD diagnosis	

#### 2.1.2 Exclusion criteria

(1) Inability to adequately expose the pupil or with blindness, strabismus and congenital spontaneous nystagmus; (2) Perforated eardrum, foreign body in the external auditory canal; (3) Other causes of cognitive dysfunction (such as Alzheimer’s disease, Lewy body dementia, vascular dementia, Parkinson’s disease, etc.); (4) Other vestibular disorders, such as benign paroxysmal positional vertigo (BPPV), vestibular neuritis, and Ménière’s disease (MD); (5) Individuals with severe physical illnesses, neuropsychiatric disorders and systemic diseases, such as stroke, coronary artery disease, schizophrenia; (6) Individuals with substance abusers; (7) Pregnant or breastfeeding women; (8) Individuals who refuse to sign the informed consent form.

### 2.2 Outcomes

(1) Demographic information: age, sex and years of education; (2) Clinical characteristics: duration of the disease and the frequency of episodes; (3) Vestibular function-related indicators: bedside and laboratory examinations (Tables 2, 3); (4) Scale scores of ACE-R, DHI, HADS, PHQ-9 and GAD-7 (Table 4).

**TABLE 2:** Items of bedside examination for vestibular function.

Head-shaking test:	The subject leaned their head forward by 20–30°. The examiner shook the subject’s head from side to side for 15 s and then observed the eye movements after stopping the shaking. If the subject exhibited horizontal, vertical, or rotational nystagmus, it was considered abnormal (positive). If no nystagmus was observed, it was considered normal (negative) (Deng et al., 2023).
Head-impulse test:	The examiner sat face-to-face with the subject and instructed the subject to fix their gaze on the examiner’s nose while keeping their neck relaxed. The examiner held the subject’s head with both hands and quickly turned the head to one side (10–20°),then observed the eye movements. If the subject’s eyes could not maintain fixation on the target and exhibited a catch-up saccade when the head was quickly moved to one side, it was considered abnormal (positive). If the subject’s eyes smoothly followed the target without a catch-up saccade, it was considered normal (negative) (Korda et al., 2020).
Test of Skew:	The subject looked straight ahead and the examiner observed the subject’s eye position during different head positions (such as tilting the head forward, backward, or to the side). If the examiner observed that one of the subject’s eyes deviated upward or downward while the other eye remained in a normal position (asymmetric skew deviation), it was considered abnormal (positive). If no skew deviation was observed (symmetric eye position), it was considered normal (negative) (Koukoulithras et al., 2022).
Romberg test:	The subject stood with their feet together, extended their arms forward, and then closed their eyes. The examiner observed the subject’s posture. If the subject exhibited significant instability, swaying, or tilting, it was considered abnormal (positive). If the subject maintained a stable standing posture with their eyes closed, it was considered normal (negative) (Halmágyi and Curthoys, 2021).
Unterberger test:	The subject extended their arms forward, closed their eyes, and took 50 steps in place. The examiner observed whether the subject’s body direction deviated. If the subject’s deviation angle exceeded 45° during the test, it was considered abnormal (positive). If the deviation angle was less than 45(, it was considered normal (negative) (Taylan Cebi and Karatas, 2022).

**TABLE 3:** Items of objective neurotological testing for vestibular function.

Video Nystagmography, VNG (Négrevergne et al., 2003; Yang et al., 2020) (Spontaneous nystagmus recording, saccade test, gaze test, smooth pursuit test, optokinetic nystagmus test: the subjects sat with their head fixed in the midline position, and their eyes were approximately 1.22 meters away from the visual target)	Spontaneous nystagmus recording: The subject looked straight ahead, and the examiner observed for any spontaneous nystagmus and involuntary eye movements
	Saccade test: The subject performed rapid saccades to a red target that appears randomly on a horizontal visual target, with a speed of 350°/second to 600°/second. The saccades were continuously recorded for 40 s, and the accuracy, latency, and peak velocity of the saccades were recorded.
	Gaze test: The subject opened their eyes (fixation) and was asked to fixate on target points 30° to the left and right, respectively. The subject then closed their eyes for a few seconds (to eliminate fixation), and suddenly opened their eyes again to continue fixating on the target points. Eye movements were recorded for more than 20 s at each position. The examiner observed and recorded the gaze nystagmus.
	Smooth pursuit test: The subject’s vision followed a target that typically moved horizontally in a sinusoidal pattern at a speed of 40°per second. The examiner observes and records the shape of the horizontal smooth pursuit curves when the visual target moves at frequencies of 0.2 to 0.3 Hz, 0.4 to 0.5 Hz, and 0.6 to 0.7 Hz.
	Optokinetic nystagmus test: The subject fixates on a visual target, which is a series of continuously moving dots on a horizontal light bar screen, moving left and right at a speed of 20°/s. Each direction is recorded for 20 s. The examiner records the gain values of optokinetic nystagmus (OKN) for leftward and rightward movements, as well as the asymmetry ratio between the two sides.
	Position test: To detect whether nystagmus could be induced when the subject’s head was in different positions. The subject assumed the following head positions: sitting, supine, with the head twisted to the left and right; supine hanging head position, with the head twisted to the left and right. Each position change was performed slowly and eye movements were recorded for at least 30 s in each head position. The test were conducted with the fixation and elimination of fixation, required the subject looking straight ahead. Rapid head movements were avoided during the test.
	Positioning test: Dix-Hallpike test: the examiner supports the subject’s head with both hands, rotates it horizontally 45° to the right, and then quickly laid the subject down to a supine position with the head hanging down about 30°. Until there was no nystagmus and no vertigo symptoms, the subject was then returned to a sitting position. Each change of position was completed within 3 s, and nystagmus and vertigo were observed for 20–30 s after each change of position. Supine roll test: the subjects takes the supine position, the head was tilted forward by 30°. The head was rotated rapidly to the right side and held in that position for 1 min to observe for nystagmus and vertigo. The head was slowly restored to the midline position. Then rotated rapidly to the left side and held in that position for 1 min to observe for nystagmus and vertigo. Any change of head position should wait until nystagmus and vertigo have stopped.
Caloric test	The bithermal caloric test was performed by applying an air caloric stimulator. The subject was placed in a supine position with the head bent forward by 30°s and underwent the first irrigation while the subject was asked to close the eyes (fixation elimination) → cold air (24°C) was delivered into the external auditory canal for 30 s → after the irrigation, the subject was asked to open the eyes and start the mental arithmetic → about 10 s after the nystagmus intensity reached its maximum, the subject was asked to fixate on a stationary target for 40 s (fixation suppression test) → the target was removed about 10 s after fixation suppression → nystagmus was recorded until the response disappeared. Then, repeated the above steps to test the contralateral ear. This test should be performed on the condition of an irrigation of 50°C. In the first 10–15 s interval after the irrigation started: the presence or absence of spontaneous nystagmus was observed; 60–90 s after the irrigation: the peak nystagmus response was observed; immediately after fixation began, the presence or absence of fixation suppression was observed. Canal paresis (CP) and directional preponderance (DP) are the main evaluation metrics in caloric testing. These values, based on Jongkees’ formula, indicate the percentage loss or hyper-reaction of unilateral hypofunction. CP < 20% indicated normal horizontal semi-circular canal function, 20–40% indicated moderate horizontal semi-circular canal function reduction and >40% indicated severe horizontal semi-circular canal function reduction (Arai et al., 1996). DP is more frequently applied in vestibular disorders, where hypofunction and hyperfunction can simultaneously occur, such as MD (Molnár et al., 2023c).

**TABLE 4:** Items of scales.

Addenbrooke’s cognitive examination- revised(ACE-R)	ACE-R is used to identify mild cognitive impairment. The Chinese version of the ACE-R has been validated in the current population and shows good reliability and internal consistency. It consists of five domains: attention and orientation (18 points), memory (26 points), verbal fluency (14 points), language (26 points), and visuospatial function (16 points). The total score is 100, with lower scores indicating poorer cognitive function. The Mini Mental State Examination (MMSE) is also included within the ACE-R. Compared to the MMSE, the Chinese version of ACE-R has higher sensitivity in identifying mild cognitive impairment with a cutoff value of 85/86 (Cao et al., 2022; Fang et al., 2014)
Dizziness Handicap Inventory(DHI):	DHI is a commonly used self-perceived evaluation scale for patients with dizziness/vertigo, Chinese version of DHI has good reliability and validity (Zhang et al., 2015). It consists of 25 sub-questions, with a total score of 100. Higher scores indicate more severe symptoms. The DHI is divided into three domains: Physical (P), Emotional (E), and Functional (F). However, the scores in these domains do not individually represent the severity of dizziness symptoms. Therefore, the total DHI score was used to subjectively measures the impact of vertigo and dizziness on daily functioning and any impairments. The classification criteria are as follows: 0–30 points for mild impairment, 31–60 points for moderate impairment, and 61–100 points for severe impairment (Fei et al., 2021).
Hospital Anxiety and Depression Scale(HADS):	HADS is a self-assessment scale primarily used for screening anxiety and depression in hospital patients. It consists of 14 sub-questions, divided into two modules: Anxiety (A) and Depression (D). Each module has a total score of 21, with higher scores indicating greater severity of anxiety and depression. The classification criteria are as follows: 0–7 points for no symptoms, 8–10 points for possible presence of anxiety or depression, and 11–21 points for definite presence of anxiety or depression (Zigmond and Snaith, 1983).
Patient Health Questionnaire-9(PHQ-9):	PHQ-9 is a simple and effective self-assessment scale for depression. It consists of 9 sub-questions, with a total score of 27. Higher scores indicate greater severity of depression. The classification criteria are as follows: 0–4 points for no depression, 5–9 points for mild depression, 10–14 points for moderate depression, 15–19 points for moderately severe depression, and 20–27 points for severe depression (Kroenke et al., 2001).
Generalized Anxiety Disorder-7(GAD-7):	GAD-7 (Generalized Anxiety Disorder-7) is a scale developed by Spitzer and colleagues for screening generalized anxiety disorder over a short period and can be used to stratify the severity of anxiety. It consists of 7 sub-questions, with a total score of 21. Higher scores indicate greater severity of anxiety over the past 2 weeks. The classification criteria are as follows: 0–4 points for no anxiety, 5–9 points for mild anxiety, 10–13 points for moderate anxiety, 14–19 points for moderately severe anxiety, and 19–21 points for severe anxiety (Spitzer et al., 2006).

### 2.3 Statistical analysis

Statistical analysis was conducted using SPSS25.0. We used the Shapiro-Wilk test to perform normality testing on our data. Categorical data were presented as *n* (%), and intergroup comparisons were performed using the chi-square test or Fisher’s exact test. Continuous data that were normally distributed were presented as mean (SD), and intergroup comparisons were conducted using the independent samples *t*-test. Continuous data that were not normally distributed were presented as median (P25, P75), and the Mann-Whitney U test was used for intergroup comparisons. A *p*-value < 0.05 was considered statistically significant and | r| > 0.3 indicated a good correlation.

To assess the relationships between the variables, we conducted a correlation analysis. For normally distributed data, we used Pearson’s correlation coefficient, and for non-normally distributed data, we used Spearman’s rank correlation coefficient. To determine the statistical significance of the correlation coefficients, we used a two-tailed test with a significance level of α = 0.05. We also created scatter plots to visually represent the relationships between the variables.

To evaluate the performance of the diagnostic tests and determine the optimal cut-off values, we performed a Receiver Operating Characteristic (ROC) curve analysis. We calculated the True Positive Rate (TPR) and False Positive Rate (FPR) for each diagnostic metric at different cut-off values. We plotted the ROC curves with FPR on the x-axis and TPR on the y-axis. The Area Under the Curve (AUC) reflected the overall performance of the diagnostic tests, AUC > 0.5 indicates that the model has better predictive performance than random guessing. The ROC curves and AUC values were presented in both graphical and tabular forms. We reported the optimal cut-off values, sensitivity, specificity, and their corresponding 95% confidence intervals for each diagnostic metric.

## 3 Results

### 3.1 Differences in age, gender, and years of education between the VM group and the HC group

A total of 61 patients were included in the VM group, with a median age of 47 years, consisting of 4 male and 57 female patients, and the median years of education for the VM group was 15 years. A total of 30 subjects were included in the HC group, with a median age of 45 years, consisting of 4 male and 26 female patients, and the median years of education was 13.5 years. There were no statistically significant differences in the general demographic data between the two groups (*p*-value > 0.05) (Table 5). Therefore, they were comparable in the subsequent studies.

**TABLE 5:** Comparison of general demographic data between VM and HC groups.

	VM(*N* = 61)	HC(*N* = 30)	χ^2^/Z	*p*-value
Age	47(37,58.5)	45(35.75,59)	−0.291	0.771
Sex			0.461	0.497
Male	4(6.6%)	4(13.3%)		
Female	57(93.4%)	26(86.7%)		
Years of education	15(12,15)	13.5(12,16)	−0.061	0.951

*p*-value < 0.05. VM, vestibular migraine; HC, healthy control; χ^2^, The chi-square test statistic; Z, the standardized position of the U statistic in the standard normal distribution in the Mann-Whitney U test; *p*-value, Significance level from the statistical test. Statistical Tests Used: Mann-Whitney U test for continuous variables, Chi-square test for categorical variables.

### 3.2 Differences in DHI scales between the VM group and the HC group

**FIGURE 1:**
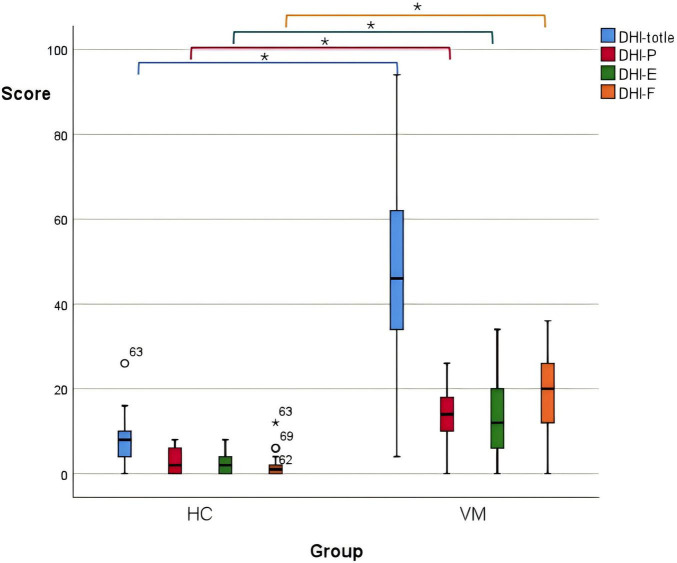
Boxplot comparing DHI scores between the VM and the HC groups. Black lines in the boxes: median values, box: the middle 50% of the data, whiskers: upper and lower 25%. * Represents extreme outliers.

There were statistically significant differences in the total DHI scores (*p*-value = 0.000), DHI-P (*p*-value = 0.000), DHI-E (*p*-value = 0.000), and DHI-F (*p*-value = 0.000) scores between the VM group and the HC group p-value (Figure 1 and Table 6). Specifically, the VM group had significantly higher DHI total score [46 (33, 63)], DHI-P [14 (10, 18)], DHI-E [12 (6, 20)], and DHI-F [20 (12, 27)] compare to the HC group, which had DHI total score [8 (4, 10.5)], DHI-P [2 (0, 6)], DHI-E [2 (0, 4)], and DHI-F [1 (0, 2.5)].

**TABLE 6:** Comparison of DHI scores between VM and HC groups.

	VM(*N* = 61)	HC(*N* = 30)	*Z*	*p*-value
DHI-total	46(33,63)	8(4,10.5)	−7.136	0.000*
DHI-P	14(10,18)	2(0,6)	−6.821	0.000*
DHI-E	12(6,20)	2(0,4)	−5.605	0.000*
DHI-F	20(12,27)	1(0,2.5)	−7.220	0.000*

*p-value < 0.05. VM: Vestibular migraine, HC: Healthy control, Z: The standardized position of the U statistic in the standard normal distribution in the Mann-Whitney U test, p-value: Significance level from the statistical test, DHI-total, the total score of Dizziness Handicap Inventory total score; DHI-P, the Physical subscale score of the Dizziness Handicap Inventory; DHI-E, the Emotional subscale score of the Dizziness Handicap Inventory; DHI-F, the Functional subscale score of the Dizziness Handicap Inventory. Statistical Tests Used: Mann-Whitney U test for continuous variables

**FIGURE 2:**
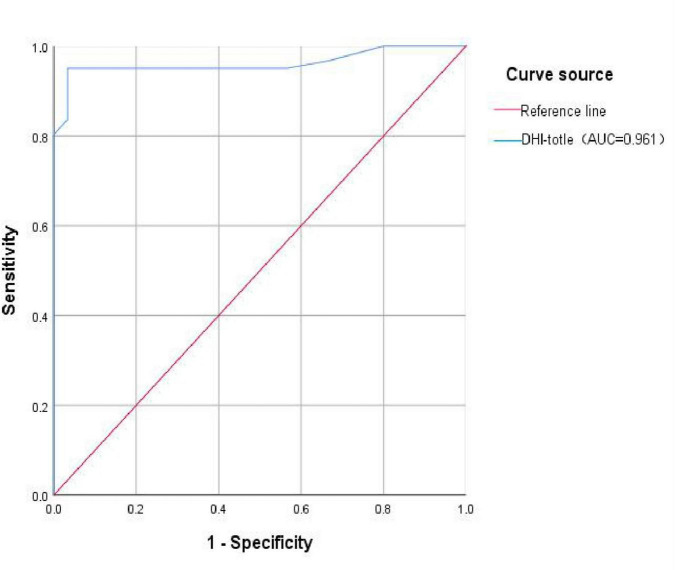
ROC curve of VM predicted by DHI total score. DHI-total, the total score of Dizziness Handicap Inventory total score; AUC, the Area Under the Curve.

Further, the ROC curve for the total DHI score in diagnosing VM was plotted. The AUC was 0.961 (95% CI: 0.922–1.000), with a corresponding p-value of 0.000, which is less than 0.05, indicating that the total DHI score has a high predictive value for VM. The cut-off value was 17, with a sensitivity of 0.951, and a specificity of 0.967, potentially serving as a predictive indicator (Figure 2 and Table 7).

**TABLE 7:** ROC curve for the total DHI score in diagnosing VM.

	AUC(95%CI)	*p*-value	Cut-off value	Sensitivity	Specificity
DHI-total*	0.961 (0.922–1.000)	0.000	17	0.951	0.967

*p-value < 0.05; CI, confidence interval; AUC, area under the curve. DHI-total, the total score of Dizziness Handicap Inventory total score; *p*-value: Significance level from the statistical test.

### 3.3 Differences in emotional scales (HADS, PHQ-9, GAD-7) between the VM and HC groups

**FIGURE 3:**
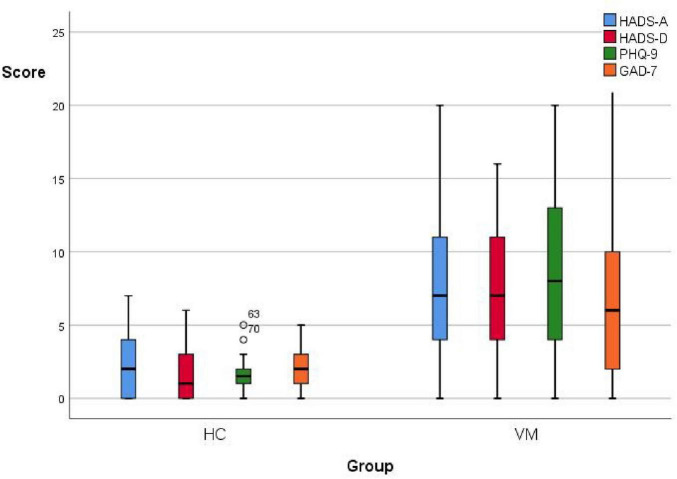
Boxplot comparing emotional scales scores between the VM and the HC groups. Black lines in the boxes: median values, box: the middle 50% of the data, whiskers: upper and lower 25%.

The emotional scales showed statistical differences between the VM group and the HC group (*p*-value = 0.000) (Figure 3 and Table 8). Specifically, the VM group had significantly higher HADS-A scores [7 (4, 11)], HADS-D scores [7 (4, 11)], PHQ-9 scores [8 (4, 13)], and GAD-7 scores [6 (2, 10)] compared to the HC group, which had HADS-A scores [2 (0, 4.25)], HADS-D scores [1 (0, 3)], PHQ-9 scores [1.5 (1, 2)], and GAD-7 scores [2 (1, 3.25)]. These results emphasized that the co-occurrence of psychiatric symptoms, particularly depression and anxiety, was significantly higher in VM patients.

**TABLE 8:** Comparison of emotional scales between VM and HC groups.

	VM(*N* = 61)	HC(*N* = 30)	*Z*	*p*-value
HADS-A	7(4,11)	2(0,4.25)	−4.974	0.000*
HADS-D	7(4,11)	1(0,3)	−5.789	0.000*
PHQ-9	8(4,13)	1.5(1,2)	−6.386	0.000*
GAD-7	6(2,10)	2(1,3.25)	−3.637	0.000*

*p-value < 0.05. VM, Vestibular migraine; HC, Healthy control; Z, The standardized position of the U statistic in the standard normal distribution in the Mann-Whitney U test, *p*-value: Significance level from the statistical test; HADS-A, Hospital Anxiety and Depression Scale-Anxiety subscale score; HADS-D, Hospital Anxiety and Depression Scale-Depression subscale score; PHQ-9, Patient Health Questionnaire-9 score; GAD-7, Generalized Anxiety Disorder-7 score. Statistical Tests Used: Mann-Whitney U test for continuous variables.

### 3.4 Differences in vestibular function between the VM and HC groups

It was statistically different in vestibular function indicators between the VM group and the HC group (*p*-value < 0.05) (Table 9). During the caloric test, we observed that VM patients exhibited reduced nystagmus intensity or even no nystagmus in response to cold/warm stimuli, as well as asymmetry in the intensity of nystagmus bilaterally. Additionally, some VM patients showed a change in the direction of nystagmus, such as nystagmus instead directed to the left when the right ear was stimulated with warm water. Specifically, the VM group had significantly higher percentages than the HC group for canal paresis (82.0% vs. 3.3%), oculor motor dysfunction (49.2% vs. 0.0%), positive head-shaking test (27.9% vs. 0.0%), positive head-impulse test (37.7% vs. 0.0%), positive Romberg’s sign (60.7% vs. 0.0%), and positive Unterberger’s sign (60.7% vs. 0.0%). Since the results of the test of skew were negative for all subjects in both groups, no statistical analysis was performed for this test.

**TABLE 9:** Comparison of vestibular function between VM and HC groups.

	VM(*N* = 61)	HC(*N* = 30)	χ^2^	*p*-value
Canal paresis	50(82.0%)	1(3.3%)	50.476	0.000*
ocular motor dysfunction-nystagmus*	30(49.2%)	0(0.0%)	22.010	0.000*
Head-shaking test (+)	17(27.9%)	0(0.0%)	10.281	0.001*
Head-impulse test (+)	23(37.7%)	0(0.0%)	15.137	0.000*
Romberg test (+)	37(60.7%)	0(0.0%)	30.665	0.000*
Unterberger test (+)	37(60.7%)	0(0.0%)	30.665	0.000*

*p-value < 0.05; (+) means one test showed positive results. VM, Vestibular migraine, HC, Healthy control, χ^2^The chi-square test statistic, *p*-value: Significance level from the statistical test. Statistical Tests Used: Chi-square test for categorical variables.

### 3.5 Differences in ACE-R between the VM and HC groups

The differences in the ACE-R total score, memory, verbal fluency, language, and visuospatial function scores between the VM group and the HC group were statistically significant (*p*-value < 0.05), while the differences in MMSE scores, attention, and orientation scores were not (*p*-value > 0.05) (Table 10). Specifically, the ACE-R total score [82 (68.5, 87)], memory score [18 (15, 24)], verbal fluency score [8 (6, 10)], language score [22 (19.5, 25)], and visuospatial functioning score [15 (12.5, 16)] in the VM group were significantly lower than those in the HC group, which had ACE-R total score [90.5 (86, 96)], memory score [24 (20, 25)], verbal fluency score [11 (9.75, 13)], language score [24 (23, 25)], and visuospatial function score [16 (14.75, 16)].

**TABLE 10:** Comparison of cognitive function between VM and HC groups.

	VM(*N* = 61)	HC(*N* 30)	*Z*	*p*-value
ACE-R total score	82(68.5,87)	90.5(86,96)	−5.277	0.000*
MMSE score	28(26,29)	28(27,29.25)	−0.539	0.590
Attention and orientation score	17(16,18)	17(16,18)	−0.199	0.842
Memory score	18(15,24)	24(20,25)	−4.078	0.000*
Verbal fluency score	8(6,10)	11(9.75,13)	−4.974	0.000*
Language score	22(19.5,25)	24(23,25)	−2.732	0.006*
Visuospatial function score	15(12.5,16)	16(14.75,16)	−3.125	0.002*

*p-value < 0.05. VM, Vestibular migraine; HC, Healthy control; Z, The standardized position of the U statistic in the standard normal distribution in the Mann-Whitney U test, *p*-value: Significance level from the statistical test, ACE-R total score, the total score of the Chinese version the Addenbrooke’s cognitive examination- revised; MMSE score, the score of the Mini Mental State Examination. Statistical Tests Used: Mann-Whitney U test for continuous variables.

### 3.6 Analysis of factors influencing VM-related cognitive dysfunction

#### 3.6.1 Comparison of general demographic data, clinical manifestations, vestibular function tests, and scale results between the VM-CogD group and the VM-NoCogD group

It can be seen that only the differences in CP value (*p*-value = 0.000), age (p-value = 0.012), years of education (p-value = 0.016), and course of the disease (*p*-value = 0.045) were statistically different between the two groups, There were no statistical differences (*p*-value > 0.05) in sex, frequency of episodes, total DHI score, DHI-P, DHI-E, DHI-F, HADS-A, HADS-D, PHQ-9, GAD-7 scores, percentage of canal paresis and ocular motor dysfunctions, positive percentage of head-shaking test, head-impulse test, Romberg test, Unterberger test (Table 11). However, there was a significant unequal sex distribution in both groups, with a notably higher proportion of females than males. This seems to validate the observation mentioned in the introduction that VM is more common in females. Specifically, the VM-CogD group had significantly higher CP values (34.14 ± 18.051) and age [51 (40, 60)] compared to the VM-NoCogD group (CP: 12.28 ± 10.016, age: 36 (25, 57.5)). The years of education [12 (12, 15)] were significantly lower, and the disease duration [8 (5, 10)] was significantly longer in the VM-CogD group compared to the VM-NoCogD group (education: 15 (12, 16), disease duration: 5 (2, 8.5)).

**TABLE 11:** Comparison of multiple factors between the VM-CogD and the VM-NoCogD groups.

	VM-CogD group(*N* = 43)	VM-NoCogD group(*N* = 18)	χ^2^ /t/Z /Fisher exact test	*p*-value
Age	51(40,60)	36(25,57.5)	−2.524	0.012*
Sex			0.595	0.440
Male	4(9.3%)	0(0.0%)		
Female	39(90.7%)	18(100.0%)		
Years of education	12(12,15)	15(12,16)	−2.403	0.016*
DHI total	46.60 ± 20.901	47.78 ± 22.259	−0.196	0.845
DHI-P	14.51 ± 6.533	13.56 ± 5.113	0.553	0.582
DHI-E	12.72 ± 8.461	13.56 ± 10.461	−0.327	0.745
DHI-F	19.37 ± 9.720	20.11 ± 9.542	−0.272	0.786
HADS-A	7.37 ± 4.337	8.00 ± 5.851	−0.464	0.644
HADS-D	7(4,11)	9(2.75,11)	−0.349	0.727
PHQ-9	8.00 ± 4.551	9.22 ± 6.274	−0.748	0.461
GAD-7	6(2,10)	6(2,10.75)	−0.087	0.930
CP value(%)	34.14 ± 18.051	12.28 ± 10.016	4.822	0.000*
ocular motor dysfunction-nystagmus	24(55.8%)	6(33.3%)	2.566	0.109
Head-shaking test (+)	12(27.9%)	5(27.8%)	0.000	0.992
Head-impulse test (+)	18(41.9%)	5(27.8%)	1.071	0.301
Romberg test (+)	28(65.1%)	9(50.0%)	1.215	0.270
Unterberger test (+)	27(62.8%)	10(55.6%)	0.278	0.598
Course of disease	8(5,10)	5(2,8.5)	−2.006	0.045*
Frequency of seizures			14.897	0.212
1 time /2 months	2(4.7%)	0(0.0%)		
1 time/ month	9(20.9%)	2(11.1%)		
1–2 times/ month	9(20.9%)	6(33.3%)		
2–3 times/ month	1(2.3%)	1(5.6%)		
3–4 times/ month	3(7.0%)	0(0.0%)		
4–5 times/ month	1(2.3%)	0(0.0%)		
1–2 times/ year	3(7.0%)	6(33.3%)		
1–3 times/ year	1(2.3%)	0(0.0%)		
2–3 times/ year	2(4.7%)	0(0.0%)		
3–4 times/ year	3(7.0%)	3(16.7%)		
4–5 times/ year	6(14.0%)	0(0.0%)		
5 times/ year	1(2.3%)	0(0.0%)		
5–6 times/ year	1(2.3%)	0(0.0%)		
6–7 times/ year	1(2.3%)	0(0.0%)		

*p-value < 0.05, (+) means one test showed positive results. VM-CogD, Vestibular migraine with cognitive dysfunction; VM-NoCogD, Vestibular migraine without cognitive dysfunction, χ^2^The chi-square test statistic, t, the independent samples *t*-test statistic, Z: The standardized position of the U statistic in the standard normal distribution in the Mann-Whitney U test, *p*-value: Significance level from the statistical test, DHI-total, the total score of Dizziness Handicap Inventory total score; DHI-P, the Physical subscale score of the Dizziness Handicap Inventory; DHI-E, the Emotional subscale score of the Dizziness Handicap Inventory; DHI-F, the Functional subscale score of the Dizziness Handicap Inventory; HADS-A, Hospital Anxiety and Depression Scale-Anxiety subscale score; HADS-D, Hospital Anxiety and Depression Scale-Depression subscale score; PHQ-9, Patient Health Questionnaire-9 score; GAD-7, Generalized Anxiety Disorder-7 score; CP value, Canal Paresis value. Statistical Tests Used: Chi-square test and Fisher exact test for categorical variables, The independent samples t-test and Mann-Whitney U test for continuous variables.

#### 3.6.2 Correlation analysis of cognitive function with CP value, age, years of education, and disease duration in the VM-CogD group

**FIGURE 4:**
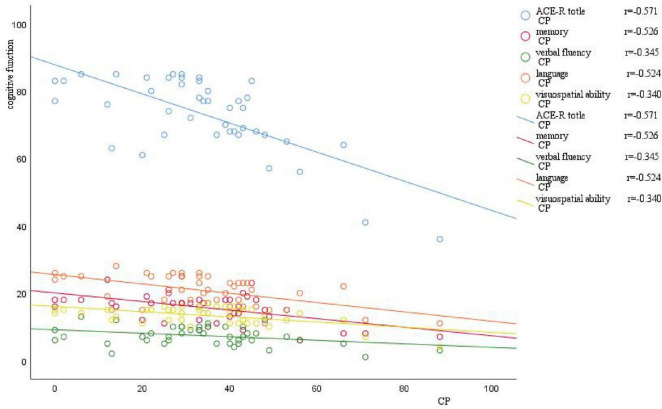
Scatter plot of correlation between CP value and cognitive function. The horizontal axis represents the CP value, and the vertical axis represents cognitive function. As the CP value increases, there is a varying degree of decrease in different domains of cognitive function.

There was a significant negative correlation between the ACE-R total score (*r* = −0.571, *p*-value = 0.000), memory (*r* = −0.526, *p*-value = 0.000), verbal fluency (*r* = −0.345, *p*-value = 0.024), language (*r* = −0.524, *p*-value = 0.000), visuospatial function (*r* = −0.340, *p*-value = 0.026) scores and the CP values (Table 12). A significant negative correlation was also observed between language (*r* = −0.384, *p*-value = 0.011) and age. Moreover, the ACE-R total score (*r* = 0.504, *p*-value = 0.001) and the language skill score (*r* = 0.455, *p*-value = 0.002) was positively correlated with years of education (Figure 4).

**TABLE 12:** Correlation analysis of cognitive dysfunction.

	ACE-R		Memory		Verbal fluency		Language		Visuospatial function	
	***r***	***p*-value**	***r***	***p*-value**	**r**	***p*-value**	***r***	***p*-value**	***r***	***p*-value**
CP value	−0.571*	0.000*	−0.526*	0.000*	−0.345*	0.024*	−0.524*	0.000*	−0.340*	0.026*
DHI total	0.074	0.636	−0.027	0.863	0.057	0.719	0.112	0.475	0.195	0.209
HADS-A	0.070	0.657	−0.035	0.822	−0.203	0.191	0.166	0.287	0.273	0.077
HADS-D	−0.165	0.290	−0.085	0.586	−0.284	0.065	−0.139	0.374	0.102	0.517
PHQ-9	0.078	0.618	−0.079	0.615	−0.052	0.742	0.172	0.270	0.131	0.404
GAD-7	0.069	0.658	0.124	0.428	−0.084	0.590	0.122	0.436	0.164	0.293
Age	−0.30	0.050	0.062	0.694	−0.198	0.202	−0.384*	0.011*	−0.064	0.683
Course of disease	−0.035	0.821	0.297	0.053	−0.017	0.912	−0.200	0.199	0.048	0.759
Years of education	0.504*	0.001*	0.202	0.193	0.247	0.110	0.455*	0.002*	0.206	0.185

*P < 0.05; *, | r| > 0.3. *p*-value: Significance level from the statistical test, r: Correlation coefficient, CP value, Canal Paresis value; DHI-total, the total score of Dizziness Handicap Inventory total score; HADS-A, Hospital Anxiety and Depression Scale-Anxiety subscale score; HADS-D, Hospital Anxiety and Depression Scale-Depression subscale score; PHQ-9, Patient Health Questionnaire-9 score; GAD-7, Generalized Anxiety Disorder-7 score; ACE-R, the total score of the Chinese version the Addenbrooke’s cognitive examination- revised. Statistical Tests Used: Pearson’s correlation coefficient for normally distributed data, and Spearman’s rank correlation coefficient for non-normally distributed data

#### 3.6.3 Predictive analyses of CP value, age, years of education, and disease duration of VM for VM-related cognitive dysfunction

**FIGURE 5:**
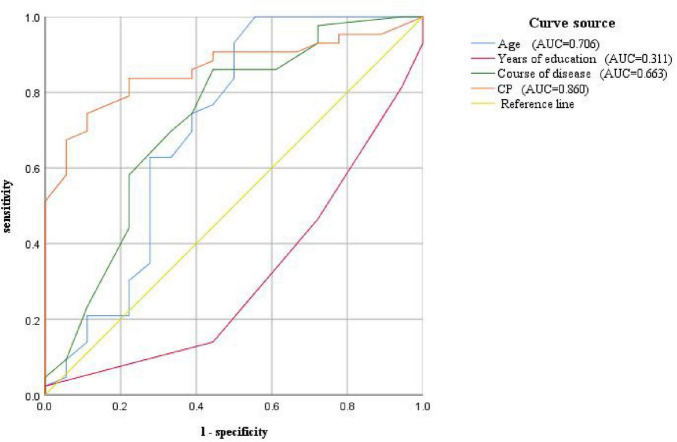
ROC curve for predicting VM-related cognitive impairment based on CP value, age, years of education, and disease course

We further drew ROC curves for VM-related cognitive dysfunction using CP value, age, years of education, and disease duration of VM (Figure 5) The AUC values were 0.860 (95% CI: 0.769–0.952), 0.706 (95% CI: 0.537–0.875), 0.311 (95% CI: 0.164–0.457), 0.663 (95% CI: 0.512–0.813), respectively. The corresponding p-values were 0.000, 0.012, 0.021, and 0.046. This indicated that the CP value, age, years of education, and duration of the VM disease had a certain predictive value for the cognitive dysfunction in VM, with cut-off values of 25.5, 33, 15.5, and 6.5, respectively (Table 13). Therefore, the diagnosis of VM-related cognitive dysfunction could be predicted when the CP value was greater than 25.5%, age was older than 33 years, years of education is less than 15.5 years, and disease duration of VM is longer than 6.5 years.

**TABLE 13:** ROC curve of cognitive dysfunction related to VM diagnosis based on CP value, age, years of education, and disease course.

	AUC(95%CI)	*p*-value	Cut-off value	Sensitivity	Specificity
CP value	0.860 (0.769–0.952)*	0.000*	25.5	0.744	0.889
Age	0.706 (0.537–0.875)*	0.012*	33	1.000	0.444
Years of education	0.311 (0.164–0.457)	0.021*	15.5	0.444	0.860
Course of disease	0.663 (0.512–0.813)*	0.046*	6.5	0.581	0.722

*p-value < 0.05; AUC > 0.5, CI, confidence interval; AUC, area under the curve; CP value, Canal Paresis value.

#### 3.6.4 Analysis of the impact of DHI, HADS, PHQ-9, and GAD-7 scale scores on VM-related cognitive dysfunction

There was no statistically significant difference in the DHI scale and anxiety/depression scales between the VM-CogD group and the VM-NoCogD group. Further subgroup analysis based on the total DHI score in VM-CogD revealed that the majority (23) of the 43 patients with VM-CogD exhibited moderate dizziness/vertigo. Patients with moderate dizziness/vertigo showed a negative correlation between ACE-R total score and the HADS-D score (*p*-value = 0.047), between memory score and the DHI total score (*p*-value = 0.008), the DHI-E score (*p*-value = 0.016), the HADS-D (*p*-value = 0.023), between language score and the DHI-F score (*p*-value = 0.031), between visuospatial ability score and the DHI-F score (*p*-value = 0.049) (Table 14). VM patients’ cognitive function was significantly lower in those with concomitant depression, vestibular dysfunction, and more severe dizziness. The correlation between DHI, HADS, PHQ-9, and GAD-7 scores and cognitive dysfunction was lower in patients who exhibited mild and severe dizziness/vertigo (| r| < 0.3). In patients with VM-CogD, those who exhibited moderate dizziness/vertigo showed the strongest correlation between CP values and ACE-R total score. Furthermore, the CP values were negatively correlated with memory and language function in patients with moderate dizziness/vertigo and memory in patients with severe dizziness/vertigo (Tables 15, 16).

**TABLE 14:** Correlation analysis of VM-CogD presenting with moderate dizziness/vertigo.

	ACE-R		Memory		Verbal fluency		Language		Visuospatial ability	
	***r***	***p*-value**	***r***	***p*-value**	***r***	***p*-value**	***r***	***p*-value**	***r***	***p*-value**
CP value	−0.757*	0.000*	−0.528*	0.010*	0.366	0.086	−0.632*	0.001*	−0.407	0.054
DHI total	−0.238	0.274	−0.539*	0.008*	−0.158	0.473	0.038	0.864	0.003	0.991
DHI-P	0.190	0.383	−0.211	0.334	−0.009	0.968	0.311	0.149	0.250	0.250
DHI-E	−0.056	0.797	−0.497*	0.016*	−0.142	0.518	0.341	0.111	0.130	0.554
DHI-F	−0.374	0.079	0.024	0.913	−0.040	0.855	−0.450*	0.031*	−0.415*	0.049*
HADS-A	0.112	0.612	−0.009	0.968	−0.202	0.357	0.219	0.316	0.045	0.838
HADS-D	−0.418*	0.047*	−0.471*	0.023*	−0.199	0.364	−0.221	0.311	−0.186	0.396
PHQ-9	0.013	0.953	−0.134	0.543	−0.159	0.469	0.300	0.164	−0.006	0.977
GAD-7	−0.055	0.804	0.013	0.953	−0.098	0.655	0.103	0.640	−0.016	0.941

*P < 0.05; *, | r| > 0.3.

**TABLE 15:** Correlation analysis of VM-CogD presenting with mild dizziness/vertigo.

	ACE-R		Memory		Verbal fluency		Language		Visuospatial ability	
	***r***	***p*-value**	***r***	***p*-value**	***r***	***p*-value**	***r***	***p*-value**	***r***	***p*-value**
CP value	−0.606*	0.048*	−0.261	0.438	−0.532	0.092	−0.421	0.197	−0.152	0.656
DHI total	0.529	0.094	0.011	0.974	0.516	0.104	0.493	0.123	0.133	0.697
DHI-P	0.233	0.491	0.018	0.958	0.267	0.427	0.170	0.617	0.041	0.905
DHI-E	−0.123	0.719	−0.251	0.457	−0.085	0.804	−0.110	0.747	0.076	0.824
DHI-F	0.533	0.092	0.149	0.661	0.751*	0.008*	0.634*	0.036*	0.340	0.306
HADS-A	−0.144	0.673	0.126	0.711	−0.340	0.306	−0.183	0.590	0.375	0.255
HADS-D	−0.085	0.803	0.068	0.843	−0.555	0.076	−0.189	0.577	0.086	0.802
PHQ-9	0.007	0.984	−0.142	0.676	−0.335	0.314	−0.174	0.609	−0.002	0.995

*P < 0.05; *, | r| > 0.3.

**TABLE 16:** Correlation analysis of VM-CogD presenting with severe dizziness/vertigo.

	ACE-R		Memory		Verbal fluency		Language		Visuospatial ability	
	***r***	***p*-value**	***r***	***p*-value**	***r***	***p*-value**	***r***	***p*-value**	***r***	***p*-value**
CP value	−0.387	0.304	−0.803*	0.009*	−0.303	0.428	−0.366	0.333	0.114	0.770
DHI total	0.080	0.838	0.389	0.301	0.137	0.726	0.080	0.838	−0.317	0.405
DHI-P	0.113	0.773	0.187	0.630	0.288	0.453	0.087	0.825	−0.217	0.575
DHI-E	0.332	0.383	0.190	0.625	0.107	0.785	0.441	0.234	−0.256	0.507
DHI-F	0.039	0.921	0.495	0.175	−0.096	0.806	−0.086	0.825	−0.203	0.601
HADS-A	−0.113	0.771	−0.503	0.168	−0.202	0.602	0.160	0.682	0.430	0.248
HADS-D	0.241	0.533	0.242	0.530	−0.075	0.848	0.283	0.461	0.582	0.100
PHQ-9	0.504	0.166	0.028	0.943	0.556	0.120	0.286	0.455	0.406	0.278
GAD-7	0.159	0.683	0.144	0.712	0.000	1	0.176	0.651	0.385	0.307

*P < 0.05; *, | r| > 0.3. *p*-value: Significance level from the statistical test, r: Correlation coefficient; CP value, Canal Paresis value; DHI-total, the total score of Dizziness Handicap Inventory total score; HADS-A, Hospital Anxiety and Depression Scale-Anxiety subscale score; HADS-D, Hospital Anxiety and Depression Scale-Depression subscale score; PHQ-9, Patient Health Questionnaire-9 score; GAD-7, Generalized Anxiety Disorder-7 score; ACE-R, the total score of the Chinese version the Addenbrooke’s cognitive examination- revised. Statistical Tests Used: Pearson’s correlation coefficient for normally distributed data, and Spearman’s rank correlation coefficient for non-normally distributed data.

## 4 Discussion

Our study found that VM, a paroxysmal vestibular disorder, involves both peripheral and central vestibular dysfunction, primarily manifesting as nystagmus and abnormalities in the caloric test, Romberg test, and Unterberger test. Although the median age of VM patients (47 years) is lower compared to Alzheimer’s Disease (AD), VM patients also exhibit cognitive dysfunction, mainly affecting visuospatial function, memory, verbal fluency, and language skill. However, the overall degree of cognitive dysfunction is relatively mild, with a median ACE-R total score of 82 out of 100(82/100). Further analysis of the factors related to VM-associated cognitive dysfunction revealed that the patient’s age, years of education, duration of VM disease, severity of vestibular dysfunction, and comorbid anxiety and depression can all influence the cognitive function of VM patients.

The caloric test is one of the main vestibular tests, alongside other important assessments. It assess the function of the bilateral horizontal semicircular canals with low-frequency stimulation, and is more sensitive to mild asymmetries in bilateral vestibular function (Mezzalira et al., 2017). CP value is a parameter generally considered for peripheral vestibular lesions, representing unilateral canal paresis (Molnár et al., 2023a). The DP value represents unilateral hyperresponse and is often used in patients with Meniere’s disease (MD), as both hypofunction and hyperfunction can occur simultaneously in this condition. Additionally, the DP value can show dynamic changes at different stages of vestibular disorders, even presenting opposite results. To ensure consistency in the results, this study only used the CP value (Lin et al., 2009). The head-shaking test is a medium-frequency stimulus, and the presence of characteristic unidirectional or bidirectional nystagmus suggests peripheral vestibular damage, and the intensity of the nystagmus can also determine the degree of vestibular compensation (Deng et al., 2023). The head-impulse test is a high-frequency stimulus. The presence of a significant catch-up saccade typically indicates an abnormality in the patient’s vestibular-ocular reflex (VOR) pathway, suggesting peripheral vestibular dysfunction (Korda et al., 2020). Romberg’s test and Unterberger’s test are based on the vestibulospinal reflex (VSR) pathway. When a patient has central vestibular lesions, these tests may reveal swaying with eyes closed and a tendency to fall or lean to one side (Halmágyi and Curthoys, 2021; Hemm et al., 2023). The combination of nystagmus test, head impulse test, and test of skew is known as the HINTS triad and is widely used to identify various acute vestibular syndromes (Kattah, 2018). Combining the HINTS triad with a bedside hearing test forms the HINTS+ test, which can effectively distinguish between acute central and peripheral vestibular lesions, particularly

in the context of posterior circulation strokes (Tarnutzer and Edlow, 2023). Alterations in the nystagmus system are numerous and complex. The advent of videonystagmography (VNG) has enabled the recording and study of subtle nystagmus that cannot be detected by the naked eye. VNG can record nystagmus caused by different lesions in the vestibular system, such as peripheral, central, or mixed (Skóra et al., 2018).

Vestibular function tests are often crucial and informative for definitive diagnosis, whether the vestibular disorder is central or peripheral. Recently, Meniere’s disease (MD) has been recognized as a cochleo-vestibular disorder rather than a mere cochlear disorder. In the early stages of MD, there is a decrease in amplitude of cervical vestibular evoked myogenic potentials (cVEMP), and in the later stages, there is a significant canal paresis in caloric testing and gain asymmetry in the video head impulse test (vHIT) (Sobhy et al., 2019). MD also presents with various ocular motor abnormalities, including spontaneous nystagmus, gaze test, saccade test, smooth pursuit, and optokinetic nystagmus in VNG. Positional nystagmus is crucial for the diagnosis of benign paroxysmal positional vertigo (BPPV), as the changes in nystagmus in different positions can identify the affected semicircular canal (Ziemska-Gorczyca et al., 2024). Additionally, patients with BPPV may exhibit abnormalities in smooth pursuit and optokinetic nystagmus. Patients with vestibular neuritis exhibit spontaneous nystagmus, which is generally horizontal-torsional, direction-fixed, and enhanced by removal of visual fixation. They also exhibit abnormal positional tests (Moideen et al., 2023) as well as lesions in the VOR and VSR pathways (Strupp et al., 2022).

Continuing research has found that vestibular disorders can manifest with cognitive impairments. The mobile tablet-based Vestibular Cognitive Assessment System (VCAS) is a system that use electronic devices to build a three-dimensional spatial structure based on cognitive questionnaires to assess visuospatial function in patients with vestibular disorders. Its tests have found that patients with vestibular dysfunction exhibit impaired spatial memory and spatial navigation function (Huang et al., 2023b). Among the above indicators reflecting vestibular function, CP value were correlated with the degree of cognitive dysfunction in VM patients. The more severe the semicircular canal damage, indicated by a higher CP value, the more severe the memory, verbal fluency, language, and visuospatial dysfunction in VM patients. Previous studies had reported a high prevalence of cognitive impairment in patients with VM and concomitant vestibular migraine and Menière’s disease (VMMD). These patients reported significantly higher frequencies of cognitive dysfunctional symptoms such as brain fog and chronic fatigue compared to those with MD alone (Chari et al., 2021). However, another study on cognitive function in VM patients reached the opposite conclusion, suggesting that the cognitive function of VM patients is normal. The latter study used the MMSE scale to assess cognitive function (Demirhan and Celebisoy, 2023). In our study, we used the ACE-R to evaluate cognitive function, which was more objective than self-reported symptoms and had higher sensitivity for mild cognitive impairment compared to the MMSE. This made our findings on cognitive impairment of VM more convincing. Other studies had evaluated immediate memory, delayed memory, language function, attention, executive function, and visuospatial abilities in VM patients and found dysfunction in all these areas. These cognitive dysfunction were also found to be correlated with latency and error frequency of anti-saccade. Our results were consistent with these findings, but our study included a more comprehensive assessment of the entire ocular motor system, recording spontaneous nystagmus, positional nystagmus, gaze-evoked nystagmus, and abnormalities in smooth pursuit (Lu et al., 2024). Liu et al. (2019) developed a new scale, the Neuro-Otologic Vestibular Instrument (NVI), to assess cognitive function in patients with vestibular disorders. They found that VM patients had cognitive dysfunction which was correlated with anxiety and depression scales. Our study also supports this finding. However, the NVI cannot be applied to healthy individuals, making it impossible to compare with them during the study. Therefore, the conclusions about the characteristics of cognitive dysfunction in VM were relatively unreliable. The experimental design of our study, which included inter-group comparisons, enhances the reliability of our conclusions (Liu et al., 2019).

How does canal paresis affect cognitive function? On one hand, it may be related to the transmission of neural pathways and the activity of brain regions. The vestibular system is a complex structure involving multiple components. The peripheral vestibular organs sense angular acceleration and linear acceleration and integrate this multisensory information, which is transmitted to the vestibular nucleus complex (VNC). The VNC integrates incoming information from vestibular, visual and proprioceptive senses, which are processed in the thalamus and cerebellum and reach the vestibular cortex, where the body responds to the information. In addition to the above structures, the VOR reflex and VSR reflex pathways are also essential for maintaining visual clarity and postural stability during movement (Aleman, 2022). The vestibular system overlaps with the cognitive system to some extent, where the vestibular cortex, thalamus, cerebellum, and basal ganglia are collectively involved in the influence of the vestibular system on cognitive functions (Hebert and Filley, 2022; Hitier et al., 2014). The vestibular cortex is a complex structure containing the cingulate gyrus, parietal cortex, hippocampus, and retrosplenial cortex. Among these, the parietal-insular vestibular cortex (PIVC) is the core structure of the vestibular cortex, which is activated to varying degrees when the vestibular system is stimulated. Various regions of the vestibular cortex have been consistently found to be involved in many processes of visuospatial function, such as the retrosplenial cortex in navigation and path integration, the anterior parietal cortex in integrating vestibular input to help distinguish self-motion from the motion of external objects, and the hippocampus in adjusting spatial orientation information in real-time via place cells, boundary cells, grid cells and head direction (HD) cells. The cerebellum and thalamus receive projections from the vestibular nerve or VNC, integration this information, and then project it to the vestibular cortex, forming a complex vestibular-cognitive network that modulates cognitive function. The basal ganglia area also receives vestibular input and participate in spatial cognitive processes. Galvanic vestibular stimulation (GVS) can affect subjects’ visuospatial function by modulating spatial representations in the hippocampus and striatum, but this modulation shows gender differences. It enhances spatial learning ability in females, and increase sensitivity to location and boundary information in males. Spatial boundary information is mostly associated with hippocampal activity, and location information is associated with striatal activity (Hilliard et al., 2019). An interesting study found that astronauts experience a diminished ability to perceive time and space, leading to incorrect estimation of time intervals and spatial distances. This spatiotemporal perception disorder is associated with the vestibular system. The vestibular system continuously integrates visual, proprioceptive, and vestibular input to accuracy of time and spatial position. The right parietal cortex, hippocampus, entorhinal cortex, which are part of the vestibular cortex are involved in the regulation of temporal and spatial cognition. In microgravity environments, the speed of the object movement decreases, and the interaction between the optokinetic stimuli and the vestibular system is reduced. In addition, microgravity reduces peripheral vestibular input, leading to functional changes in the right parietal cortex, ultimately resulting in spatiotemporal perception disorders (Navarro Morales et al., 2023).

On the other hand, it may be related to the allocation of cognitive resources. It has been proposed that the sum of an individual’s cognitive resources for vestibular and cognitive functioning is fixed. When vestibular function is impaired or the demands of vestibular tasks are increased, the body reduces its allocation of resources to cognitive functioning, resulting in varying degrees of cognitive dysfunction (Vermette et al., 2023). A recent dual-task study on patients with vestibular disorders found that when subjects were asked to complete a gait test while simultaneously executing cognitive tasks such as motor activities, calculations, and literacy, they performed poorly on the gait test. Furthermore, rapid visual information processing and paired-associate learning predicted the onset of gait deficits in subjects. Rapid visual information processing and paired-associate learning are important tests that reflect the cognitive function. The completion of vestibular task requires the coordinated use of cognitive resources, suggesting that vestibular function and cognitive processes are closely related. This relationship may be related to the fact that vestibular afferent stimuli affect the integration of information in the multisensory-motor integrating cortex of mesial temporal lobe, left posterior parietal lobe, and so on. Alzheimer’s disease (AD) is a neurodegenerative disorder characterized by progressive cognitive dysfunction. Numerous studies have found that vestibular dysfunction is more common in AD, and that loss of vestibular function increases the risk of dementia (Lim et al., 2023; Pavlou et al., 2023). The absence of bilateral cVEMPs and decreased cVEMP amplitudes are associated with an increased odds of AD (Harun et al., 2016). This may be related to the saccule mediating abnormal neuronal firing through neural pathways, leading to hippocampal atrophy, which is a pathological hallmark of AD. Based on the aforementioned findings, Huang et al. (2023a) proposed that cVEMPs may serve as a screening tool for AD. Appropriate vestibular rehabilitation exercises can improve balance and reduce fall risk in patients with cognitive impairment (Pavlou et al., 2023).

The severity of dizziness/vertigo in patients with vestibular disorders tends to correlate with the degree of cognitive dysfunction. Donaldson et al. (2021) found a negative correlation between DHI scores and scores on the Cognitive Fusion Questionnaire (CFQ) in patients with VM. Dornhoffer et al. (2021) concluded that changes in DHI scores in MD patients before and after treatment correlated with changes in CFQ scores. However, since the DHI cannot differentiate between different types of cognitive dysfunction associated with various vestibular disorders, its scores cannot currently be used as a screening tool for cognitive dysfunction (Liu et al., 2019). In our study, the average DHI score for VM patients was 46, indicating a moderate level of vertigo, which is significantly higher than that of the healthy individuals. However, we did not find a difference in DHI scores between VM-CogD group and VM-NoCogD group, which seems to indicate its limitations for the identification of cognitive dysfunction. To further investigate, we divided the VM-CogD into subgroups based on their DHI scores, categorizing them as having mild, moderate, or severe dizziness/vertigo. We then analyzed the correlation between DHI scores and cognitive dysfunction in these subgroups. We found that in the subgroup with moderate dizziness/vertigo subgroup, the functional disturbances in VM patients were associated with language and visuospatial dysfunction. This seems to suggest that VM is a functional disorder and there is potential to improve cognitive symptoms by addressing their functional abnormalities in VM patients.

The high comorbidity of affective disorders in patients with vestibular dysfunction may also contribute to the development of cognitive dysfunction. Xie et al. (2022) found that patients with dizziness/vertigo experienced more emotional problems and that anxiety and depression not only exacerbate the severity of the patient’s vertigo disorder (Feng and Zang, 2023), but also worsen the cognitive dysfunction caused by dizziness/vertigo. GAD-7 and PHQ-9 scores have been proposed as valid predictors of cognitive dysfunction in patients with vestibular dysfunction (Liu et al., 2019). In this study, among the VM patients, 35 had mild depressive disorders, 9 had moderate-to-severe depressive disorders, 25 had mild anxiety disorders, 10 had moderate-to-severe anxiety disorders. In the subgroup of VM-CogD who experience moderate dizziness/vertigo, the more depressed the patient was, the worse their memory was. Ma proposed that VM and anxiety / depression seem to be mutually influential. VM patients experience more severe anxiety and depression, and those with comorbid anxiety and depression have more severe dizziness. Anxiety and depression may contribute to the development of cognitive dysfunction in VM by affecting dizziness and sleep disorders (Ma et al., 2024). In this study, anxiety and depression were prevalent among VM patients. For example, 57% of VM patients had mild depressive disorder, 16% had moderate to severe depressive disorder, 41% had mild anxiety disorder, and 16% had moderate to severe anxiety disorder. We also found that the degree of depression was related to overall cognitive function and memory in VM-CogD with moderate dizziness/vertigo. The more severe the depression, the worse the cognitive function, particularly in memory. The correlation between emotional changes and cognitive function may be related to cognitive bias. Patients with anxiety and depression tend to focus on unpleasant or threatening information, leading to changes in the activity patterns of attention-related brain regions and a bias toward such information (Sass et al., 2014). Given the widespread comorbidity of vestibular dysfunction, cognitive impairment, and emotional abnormalities, Smith et al. (2019) proposed the concept of the “vestibular cognitive affective” syndrome, in which the vestibule is the foundation. Only by addressing the balance issues caused by vestibular dysfunction can the emotional and cognitive impairment be alleviated. The high correlation among vestibular, cognitive, and affective function may be related to a dominant hemisphere bias of the vestibular system. The greater the degree of the dominance bias, the lower the likelihood of cognitive and affective disorders (Bednarczuk et al., 2018). The molecular mechanisms may be the interactions between the vestibular system and regions involved in emotional regulation, such as the parabrachial nucleus, the locus coeruleus, and the insula, leading to changes in neurotransmitters such as 5-HT and norepinephrine (Balaban et al., 2011), ultimately leading to the comorbidity. In VM patients, venlafaxine, serotonin-norepinephrine reuptake inhibitor, significantly reduces the severity of dizziness disorders, the frequency of symptom episodes, and alleviate concomitant anxiety and depression in patients (Chen et al., 2023; Wang et al., 2020).

## 5 Conclusion

Our study emphasizes the following two points:

5.1 VM Patients exhibit multifaceted vestibular dysfunction and varying degrees of cognitive dysfunction, and cognitive function, is affected by age, duration of illness, years of education, and vestibular function;
5.2 VM is a functional disorder, and this function disturbance in conjunction with anxiety and depression, may participate in the occurrence of the development of the cognitive dysfunction in VM.

## Data Availability

The raw data supporting the conclusions of this article will be made available by the authors, without undue reservation.
